# CorMatrix Wrapped Around the Adventitia of the Arteriovenous Fistula Outflow Vein Attenuates Venous Neointimal Hyperplasia

**DOI:** 10.1038/s41598-017-14696-z

**Published:** 2017-10-30

**Authors:** Binxia Yang, Sreenivasulu Kilari, Akshaar Brahmbhatt, Deborah L. McCall, Evelyn Nieves Torres, Edward B. Leof, Debabrata Mukhopadhyay, Sanjay Misra

**Affiliations:** 1 Vascular and Interventional Radiology Translational Laboratory, Department of Radiology, Rochester, Minnesota USA; 2 Department of Biochemistry and Molecular Biology, Mayo Clinic, Rochester, Minnesota USA

## Abstract

Venous neointimal hyperplasia (VNH) at the outflow vein of hemodialysis AVF is a major factor contributing to failure. CorMatrix is an extracellular matrix that has been used in cardiovascular procedures primarily as scaffolding during surgery. In the present study, we sought to determine whether CorMatrix wrapped around the outflow vein of arteriovenous fistula (AVF) at the time of creation could reduce VNH. In mice, the carotid artery to the ipsilateral jugular vein was connected to create an AVF, and CorMatrix scaffold was wrapped around the outflow vein compared to control mice that received no scaffolding. Immunohistochemistry, Western blot, and qRT-PCR were performed on the outflow vein at 7 and 21 days after AVF creation. In outflow veins treated with CorMatrix, there was an increase in the mean lumen vessel area with a decrease in the ratio of neointima area/media + adventitia area (*P* < 0.05). Furthermore, there was a significant increase in apoptosis, with a reduction in cell density and proliferation in the outflow veins treated with CorMatrix compared to controls (*P* < 0.05). Immunohistochemical analysis revealed a significant reduction in fibroblasts, myofibroblasts, macrophages, and leukocytes with a reduction in *Tnf-α* gene expression (*P* < 0.05). In conclusion, outflow veins treated with CorMatrix have reduced VNH.

## Introduction

By the end of 2011, more than 600,000 individuals with end-stage renal disease (ESRD) had received hemodialysis and kidney transplants in the United States^1^. Vascular access is a lifeline for patients on hemodialysis. An arteriovenous fistula (AVF) is the preferred hemodialysis vascular access for these patients; however, only 60% of patients have a functional AVF at one year. Pathologically, this occurs due to venous neointimal hyperplasia (VNH), which leads to a reduction in venous stenosis and subsequent thrombosis^2^.

Hypoxia, hemodynamic shear stress, and inflammation are considered the initial factors that cause the pathophysiologic changes responsible for vascular access failure^3^. Our laboratory and others have demonstrated that myofibroblasts (α-smooth muscle actin [SMA]+) and fibroblasts (fibroblast specific protein [FSP-1]+) are present in venous neointimal hyperplasia. These cells may migrate from the adventitial layer when the vasculature is under stress. Moreover, accumulation of macrophages (CD68[+]) in the venous wall of the AVF is often noted^4^.

Extracellular matrix is being used as a patch for cardiovascular procedures, as it can have both a structural and functional role. The extracellular matrix scaffold CorMatrix is processed from porcine small intestine submucosa (Cook Biotech, West Lafayette, IN, for CorMatrix Cardiovascular, Inc, Roswell, GA). CorMatrix mainly contains type I collagen fibers and extracellular matrix components (glycosaminoglycans and glycoproteins) devoid of cells. The decellularized extracellular matrix serves as a scaffold that allows cells to reside from adjacent tissues^5^. CorMatrix biomaterials have been used in more than 80,000 cardiac procedures, including but not limited to the repair of atrioventricular valves, aneurysms involving the AVF, aortic root enlargement, and remodeling of the right atrium and superior vena cava^6–11^.

In the present study, we hypothesized that outlow veins treated with CorMatrix at the time of AVF creation would have reduced VNH formation^12,13^. The aim of the study was to assess the vascular remodeling of the outflow veins treated with CorMatrix compared to controls. Our results demonstrate that outflow veins treated with CorMatrix have reduced VNH and increased lumen vessel area compared to controls. This was accompanied with a decrease in cell proliferation, increased apoptosis, reduced fibroblast, smooth muscle cell, macrophage and leukocyte infiltration in the outflow veins wrapped with CorMatrix. Finally, there was a decrease in *Tnf-α* gene expression.

## Results

### Surgical Outcomes

**Figure 1:**
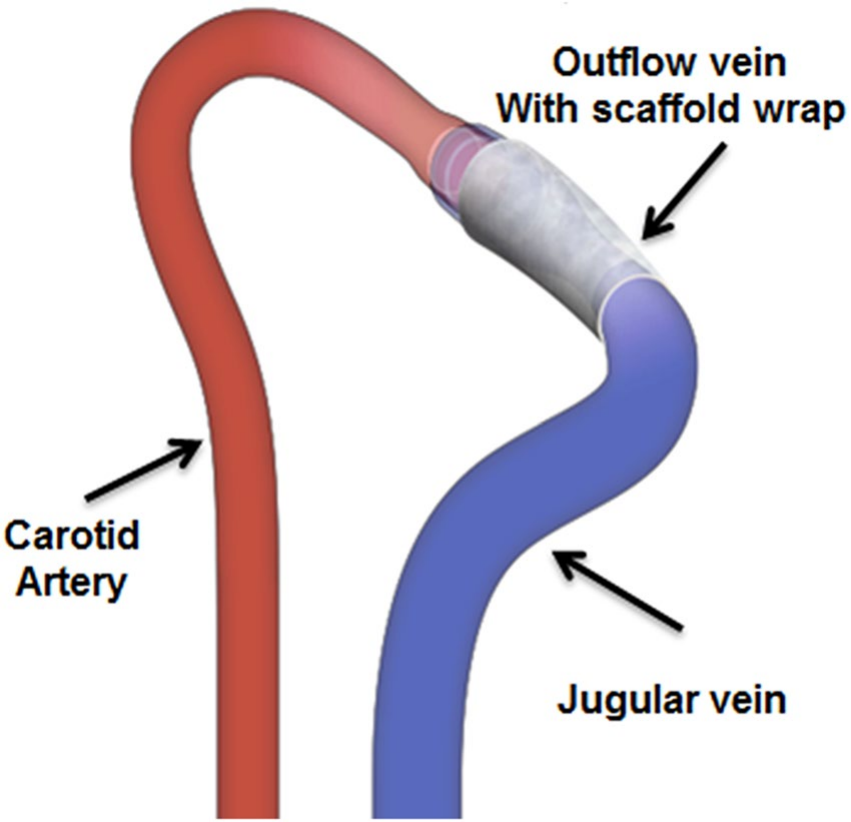
Schematic representation of an arteriovenous fistula between the carotid artery and the ipsilateral jugular vein, with a CorMatrix scaffold wrap around the outflow vein. The Mayo Clinic copyrights this figure and no changes have been made. Permission has been granted to publish this under CC BY open access license and the attribution information is available at https://creativecommons.org/licenses/by/4.0/.

Of the 46 mice, (control [Group C], n = 23; CorMatrix [Group S], n = 23), four mice expired after AVF placement (Fig. 1). The procedure had a 91% animal survival rate. QRT-PCR analysis was performed at day 7 in each group (n = 6 for both Group C and S). Histomorphometric analysis was performed at 7 and 21 days after AVF placement (n = 6 for both Group C and S). Western blot analysis was performed at day 21 (n = 3 for both Group C and S).

The AVF was evaluated for patency if the vessel filled with blood from the arterial end when the outflow vein was occluded temporarily at the distal end. By day 7, the patency rate of the AVF was 64% in Group C and 71% in Group S. By day 21, the AVF patency rate was 56% in the control group and 78% in the scaffold group (P = NS).

### Outflow veins treated with CorMatrix have a significant decrease in the average venous neointimal hyperplasia with an increase in average lumen vessel area

**Figure 2:**
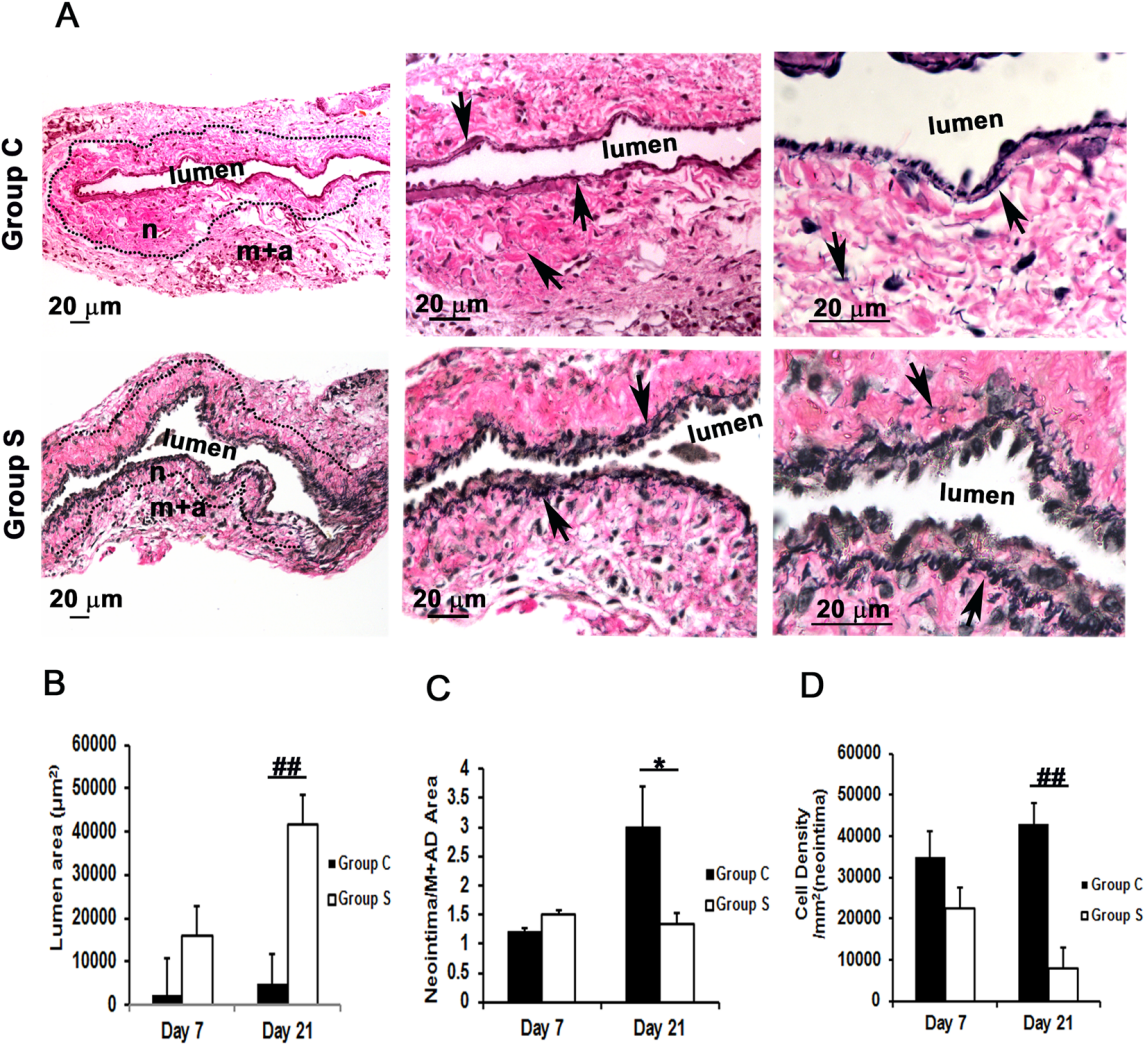
Histomorphometric analysis of outflow veins wrapped with CorMatrix scaffold. (**A**) VVG staining of outflow vein wrapped with CorMatrix (Group S) compared to controls (Group C) at day 21 after fistula creation (20×, 40×, and 100× magnifications from left to right). The boundary between the neointima and media is identified with a dotted line in the 20×-magnification image. Elastic laminae are identified with arrows in the high magnification images. m + a indicates media plus adventitia; n, neointima. (**B**) The average lumen vessel area in Group S vessels is significantly increased compared to Group C at day 21 (*P* < 0.0001). (**C**) The ratio of neointima/media + adventitia (M + Ad) is decreased in Group S compared to Group C at day 21 (*P* < 0.05). (**D**) Cell density in the neointima is decreased in Group S compared to Group C at day 21 (*P* < 0.0001). Each bar represents mean ± SEM (n = 4–6). ^*^ *P* < 0.05; ^##^ *P* < 0.0001.

Vascular remodeling of the outflow vein was determined at 7 and 21 days after AVF placement by performing histomorphometric analysis using Verhoeff-Van Gieson (VVG) staining as previously described by our laboratory^14,15^. With VVG staining, the different layers of the vessel wall could be identified including the neointima, media + adventitia, and the vascular lumen as shown (Fig. 2A). By day 21, the average lumen vessel area was significantly increased in the outflow vein from Group S compared to Group C (41, 541.1 ± 8, 919 μm^2^ vs. 5, 079.7 ± 832.5 μm^2^, respectively; average increase: 817%, *P* < 0.0001). The average ratio of neointimal area/media + adventitia area was significantly reduced in the outflow veins from Group S compared to Group C at day 21 (1.35 ± 0.18 vs. 3.0 ± 0.76, respectively; average decrease: 55%, *P* < 0.05, Fig. 2B). Hematoxylin and Eosin (H&E) staining was used to assess cell density (images not shown). The results showed the average cell density in the neointima of the outflow veins from Group S was significantly lower than Group C at day 21 (7, 990.7 ± 1, 027.3/μm^2^ vs. 43, 041.7 ± 8, 377/μm^2^, respectively; average decrease: 81%, *P* < 0.001, Fig. 2C). Next, we examined the changes in collagen 1 and 3 in the outflow veins using Picrosirius red staining. Qualitatively, this demonstrated a reduction in the intensity of Sirius red staining in Group S compared to Group C. In aggregate, this indicates that there is a decrease in constrictive remodeling of the outflow veins removed from Group S compared to Group C (Supplementary Fig. S1).

### Outflow veins treated with CorMatrix have a significant increase in TUNEL staining with reduced cellular proliferation

**Figure 3:**
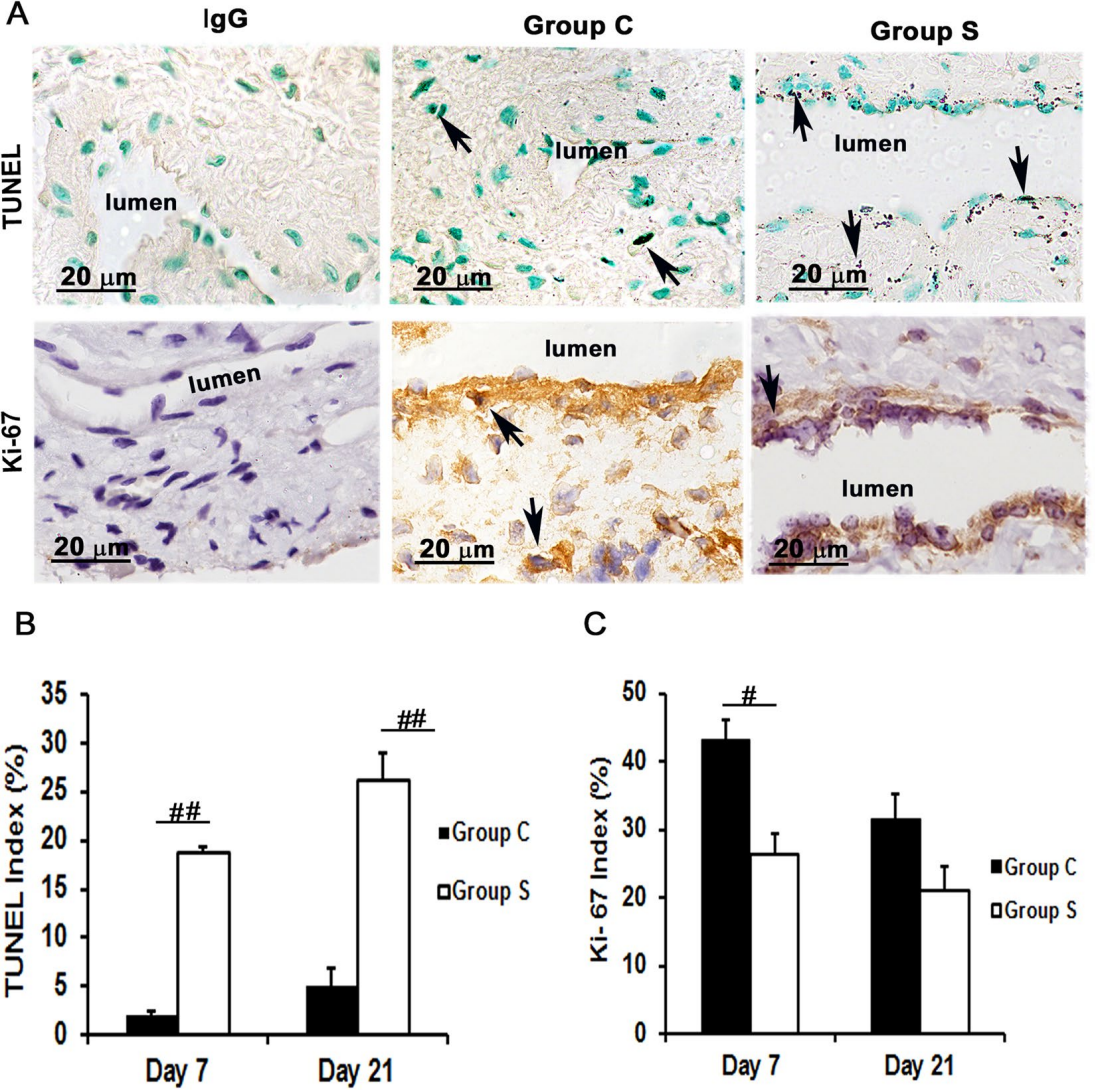
Assessment of cell apoptosis and proliferation in the AVF outflow veins wrapped with CorMatrix scaffold. Cell death and cell proliferation was assessed using TUNEL and Ki-67 staining, respectively, on outflow vein tissue sections removed at day 7 after fistula placement. (**A**) TUNEL (top row) and Ki-67 (bottom row) staining on day 7 outflow veins from control (Group C, second column) or with CorMatrix wrap (Group S, third column) are shown. The first column is the negative control (TUNEL: negative control; Ki-67: IgG). Dark brown nuclei are positive for TUNEL (top row) and brown nuclei are positive for Ki-67 (bottom row) as indicated by arrows. All images were taken at 100× magnification and the scale bar is 20 μm. (**B**) Quantitative analysis of TUNEL staining for apoptosis. Mean TUNEL staining was significantly increased in Group S compared to Group C at day 7 (*P* < 0.0001) and day 21 (*P* < 0.0001). (**C**) Quantitative analysis of Ki-67 staining for cell proliferation was performed. The average Ki-67 density was significantly reduced in Group S compared to Group C at day 7 (*P* < 0.01). Each bar represents mean ± SEM (n = 6). ^#^ *P* < 0.01; ^##^ *P* < 0.0001.

**Figure 4:**
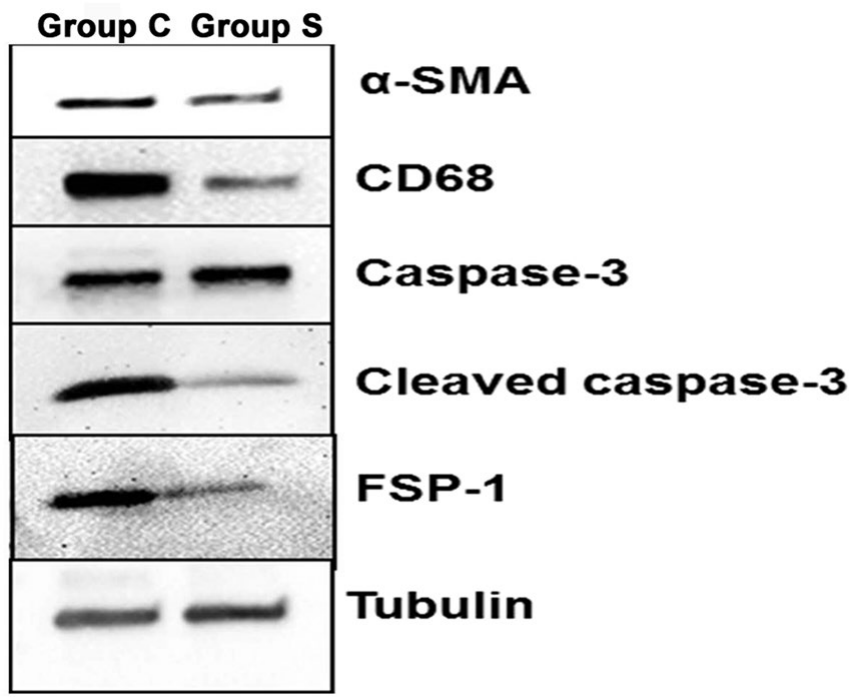
Western blot of α-SMA, CD68, caspase-3, cleaved caspase-3, and FSP-1 protein expression on day 21. α-SMA, CD68, cleaved caspase-3, and FSP-1 expression was decreased in CorMatrix-wrapped outflow veins compared with controls at day 21. Caspase-3 expression was elevated in CorMatrix-wrapped outflow vein compared with controls at day 21. Tubulin was used for loading.

We speculated that the decrease in cell density could be due to either an increase in apoptosis^16^ or a decrease in cell proliferation. Apoptosis was evaluated using Terminal deoxynucleotidyl transferase (TdT) dUTP Nick-End labeling (TUNEL) staining. The mean TUNEL (+) cells in Group S was significantly increased by day 7 (18.8 ± 1.79 vs. 1.95 ± 0.46; average increase: 962%, *P* < 0.001, Fig. 3A upper row) and remained increased at day 21 (26.1 ± 2.8 vs. 5.6 ± 0.5; average increase 516%, *P* < 0.001) when compared to Group C (Fig. 3B, Supplementary Fig. S2). To confirm these results, we measured cleaved caspase-3 levels in the outflow veins using Western blot analysis at day 21 (Fig. 4). Outflow veins removed from Group S had higher caspase-3 levels compared to Group C.

Cell proliferation was evaluated using Ki-67 staining (Fig. 3A, bottom row). There was a significant reduction in Ki-67 staining in Group S compared to Group C by day 7 (26.3 ± 1.7 vs. 43 ± 3.9; average decrease: 38.9%, *P* < 0.01, Fig. 3C). However, there was no significant difference by day 21. Together, these results suggest that outflow veins treated with CorMatrix wrap had decreased cellular proliferation at day 7 with increased apoptosis at 7 and 21 days after AVF placement.

### Outflow veins treated with CorMatrix have a significant decrease in α-SMA and FSP-1 staining

**Figure 5:**
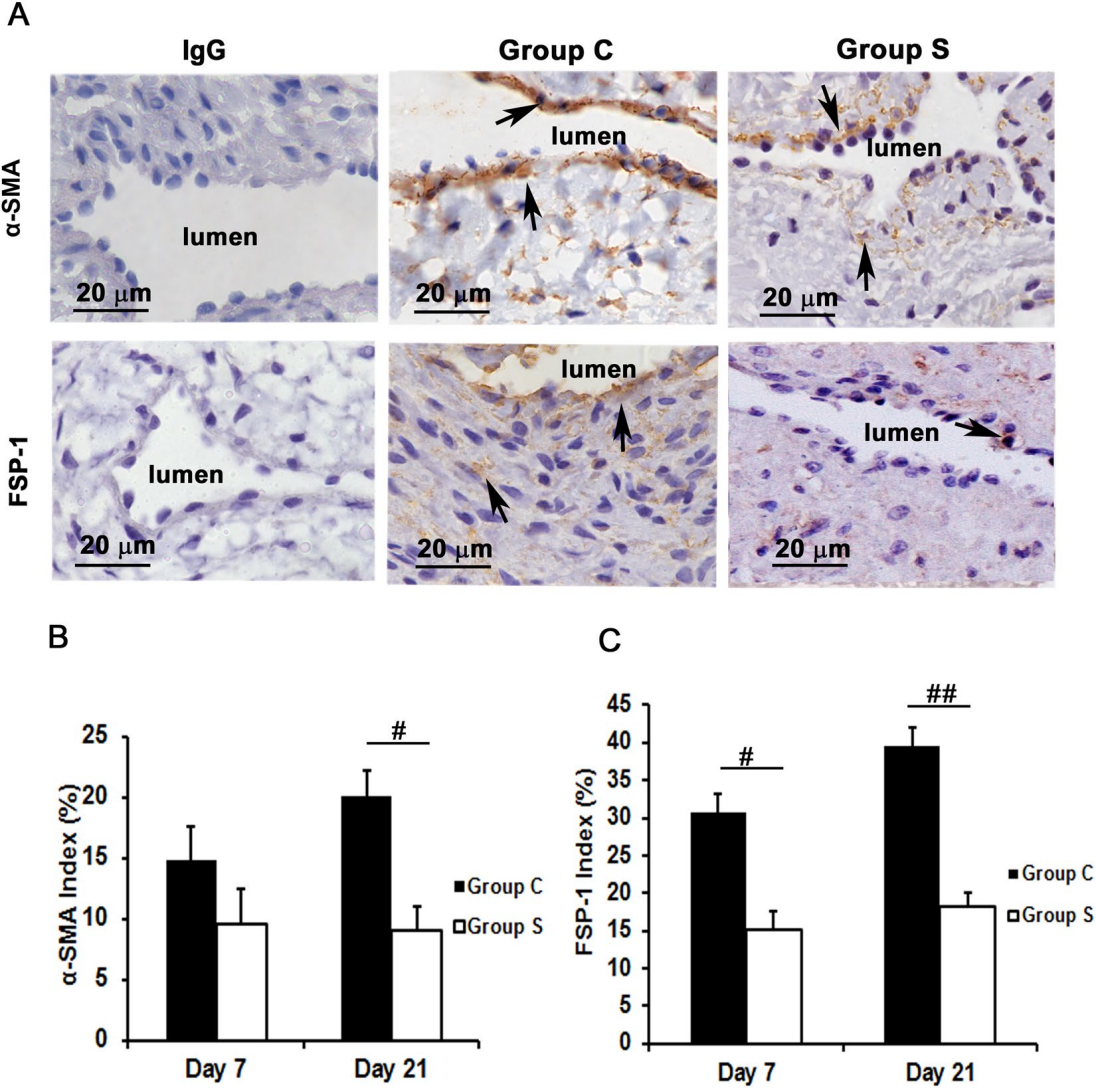
Immunostaining for fibroblast and myofibroblast in outflow veins. (**A**) Staining for α-SMA (myofibroblasts, top row) and FSP-1 (fibroblasts, bottom row) on day 21 from outflow vessels from controls (C, second column) or with (S, third column) CorMatrix wrap. The first column is the IgG antibody negative control. α-SMA or FSP-1 positive cells have brown staining cytoplasm as indicated by arrows. Images were captured at 100× magnification with the scale bar indicating 20 µm. (**B**) Quantitative analysis for α-SMA staining. The average α-SMA density is significantly reduced in Group S compared to Group C at day 21 (*P* < 0.01). (**C**) Quantitative analysis for FSP-1 staining. The average FSP-1 density is significantly reduced in Group S compared to Group C at day 7 (*P* < 0.01) and day 21 (*P* < 0.0001). Each bar represents mean ± SEM (n = 6). ^#^ *P* < 0.01; ^##^ *P* < 0.0001.

Experimental studies have shown that myofibroblasts (α-SMA [+] cells) and fibroblasts (FSP-1 [+] cells) are present in VNH of AVF^17,18^. We used immunohistochemistry to assess the presence of these cells. At day 7, the average FSP-1 (+) cells was significantly decreased in outflow veins removed from Group S compared to Group C (15.1 ± 2.3 vs. 30.6 ± 2.5; average decrease: 49.3%, *P* < 0.01, Fig. 5) and at day 21 (18.1 ± 1.9 vs. 39.4 ± 2.6; average decrease: 54.1%, *P* < 0.001, Fig. 5).

By day 21, there was a significant decrease in the average number of α-SMA (+) cells in the outflow veins removed from Group S compared to Group C (9.0 ± 1.9 vs. 20.0 ± 2.1; average decrease: 54.9%, *P* < 0.01, Fig. 5). Taken together, these results suggest that outflow vein treated with CorMatrix wrap have a significant decrease in fibroblasts and smooth muscle cells. Western blot analysis of α-SMA and FSP-1 confirmed these results (Fig. 4).

### Outflow veins treated with CorMatrix have a significant decrease in HIF-1α expression

**Figure 6:**
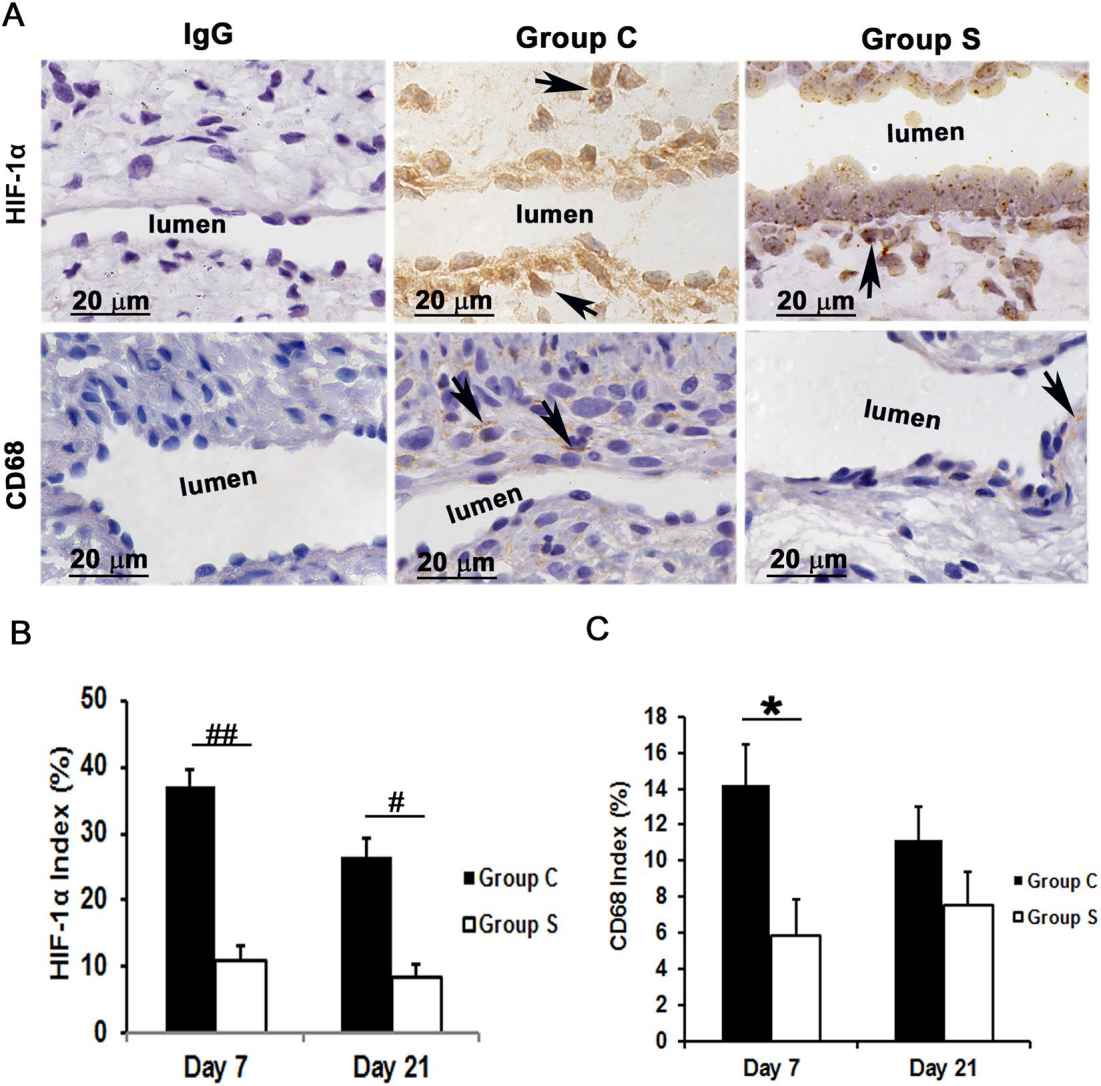
Assessment of HIF-1α and macrophage infiltration in AVF outflow veins. (**A**) Staining for HIF-1α (top row) on day 21 and CD68 (macrophages, bottom row) on day 7 from outflow vessels from controls (C, second column) or with (S, third column) CorMatrix wrap. The first column is the IgG antibody negative control. HIF-1α (+) cells have brown-staining nuclei (arrows, top row). CD68-positive macrophages have brown-staining cytoplasm (arrows, bottom row). Images were captured at 100× magnification with the scale bar indicating 20 µm. (**B**) Quantitative analysis for HIF-1α staining in the outflow vein is shown. HIF-1α staining is reduced in Group S compared to Group C at day 7 (*P* < 0.0001) and day 21 (*P* < 0.01). (**C**) Quantitative analysis for CD68 macrophage is shown. CD68 macrophages are significantly reduced in Group S compared to Group C at day 7 (*P* < 0.05). Each bar represents mean ± SEM (n = 6). ^*^ *P* < 0.05; ^#^ *P* < 0.01; ^##^ *P* < 0.0001.

Hypoxia has been implicated as one of the factors contributing to VNH^12,19^. To examine this, we performed HIF-1α staining. At day 7, we observed that the average staining density for HIF-1α was significantly reduced in outflow veins removed from Group S compared to Group C (10.8 ± 2.3 vs. 37.0 ± 2.6; average decrease: 70.6%, *P* < 0.0001, Fig. 6) and it remained lower by day 21 (8.2 ± 2.1 vs. 26.6 ± 2.4; average decrease: 68.8%, *P* < 0.01, Fig. 6).

### Outflow veins treated with CorMatrix wrap have a significant reduction in macrophage and leukocyte infiltration

**Figure 7:**
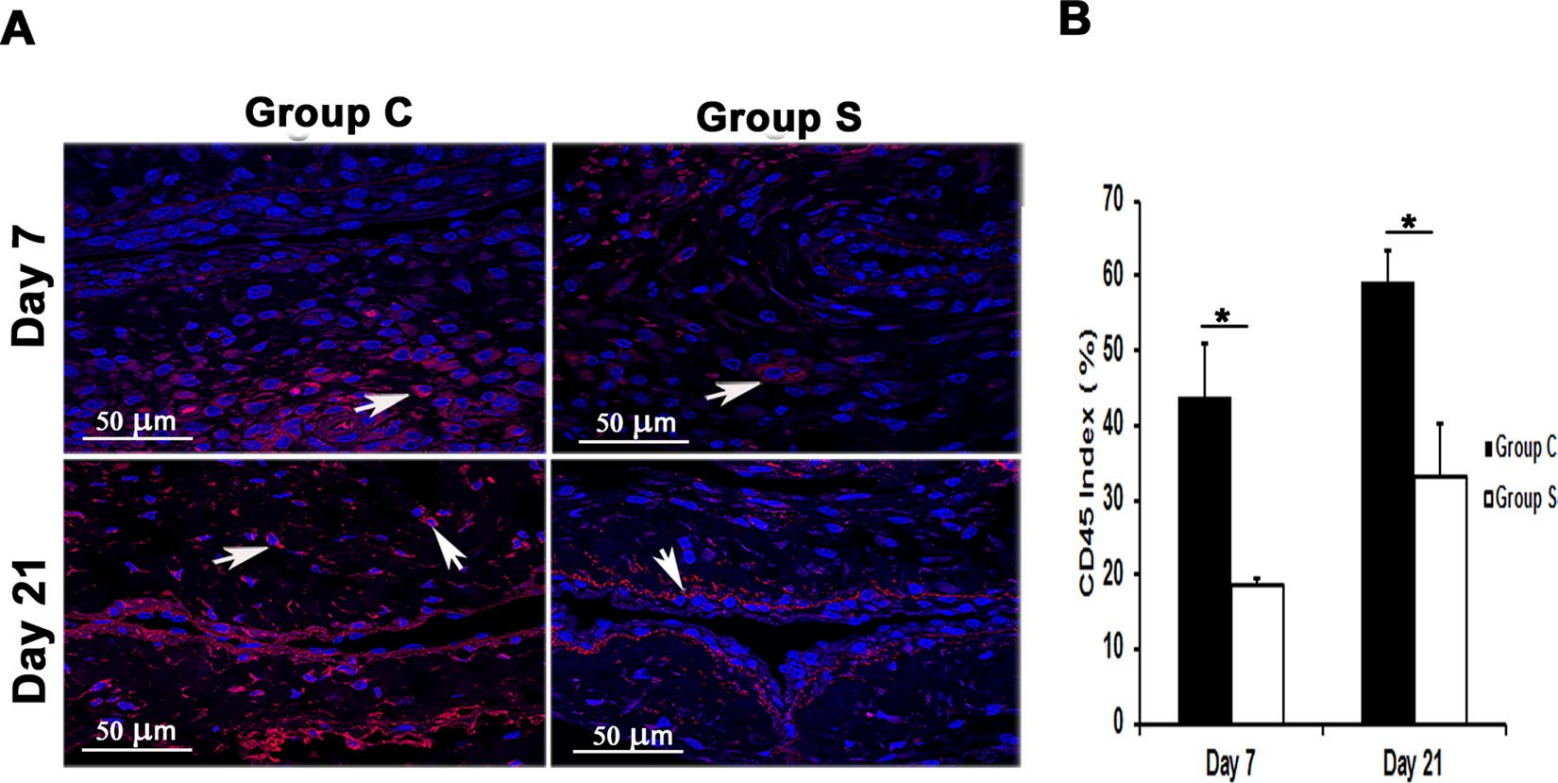
Assessment of leukocyte infiltration in the AVF outflow veins. (**A**) Staining for CD45 (leukocytes) from outflow vein of controls (C, first column) or with (S, second column) CorMatrix wrap. CD45-positive cells have cytoplasmic red staining. Images were captured at 40× magnification and the scale bar is 50 µm. (**B**) Quantitative analysis for CD45 staining in the outflow vein is demonstrated. There is a significant reduction in CD45 staining in Group S compared to Group C at day 7 (*P* < 0.05) and day 21 (*P* < 0.05). Each bar represents mean ± SEM (n = 6 per group). ^*^ *P* < 0.05.

Previous studies in clinical specimens and in pre-clinical AVF models have demonstrated that there is increased CD68 (+) cells present^12,19^. In order to assess whether CorMatrix wrap had an effect on macrophage infiltration, we stained tissue sections for CD68. By day 7, a significant decrease in the average CD68 (+) cells in outflow veins treated with Group S compared to Group C was observed (5.8 ± 2.2 vs.14.2 ± 2.3; average decrease: 58.6%, *P* < 0.05, Fig. 7). By day 21, there was a no significant difference between both groups. These results were confirmed using Western blot analysis for CD68 levels in outflow veins treated with Group S compared to Group C (Fig. 4).

Leukocyte infiltration in the vessel walls^20^ was assessed by CD45 staining. CD45 staining was significantly decreased in outflow veins from Group S compared to Group C at day 7 (18.6 ± 4.2 vs. 43.8 ± 7.2; average decrease: 57.5%, *P* < 0.05, Fig. 7) and remained significantly decreased by day 21 (33.0 ± 7.1 vs. 59.1 ± 0.9; average decrease: 49.2%, *P* < 0.05). These results suggest that outflow veins treated with CorMatrix have reduced leukocyte and macrophage infiltration.

### Outflow veins treated with CorMatrix Wrap have a significant decrease in Tnf-α gene expression

**Figure 8:**
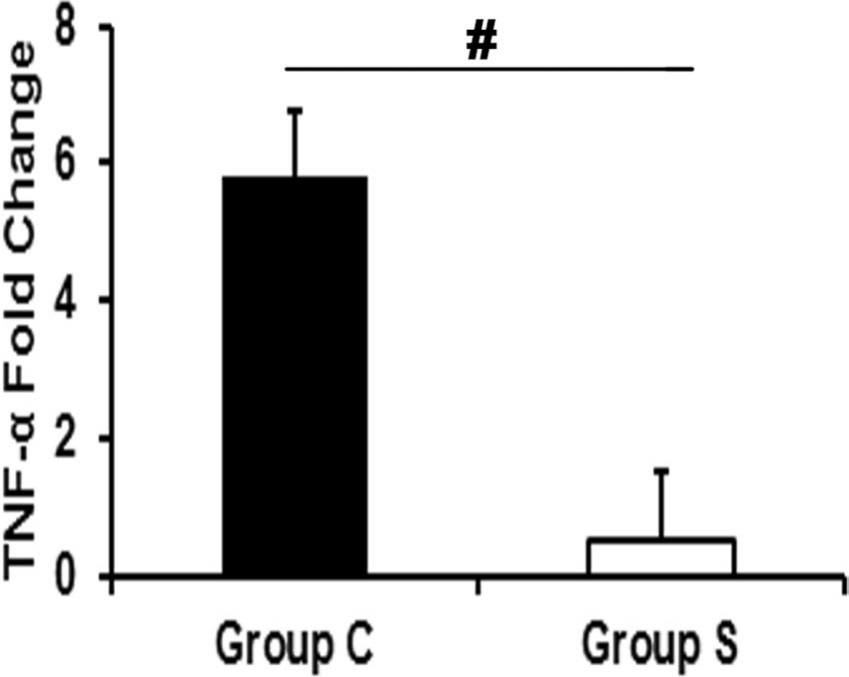
*Tnf-α* gene expression at day 7 in AVF outflow veins. *Tnf-α* gene expression using qRT-PCR was assessed and it was significantly reduced in Group S compared to Group C (*P* < 0.01). Each bar represents mean ± SEM (n = 6 per group). ^#^ *P* < 0.01.

In order to identify a mechanism for the present observations, we performed gene expression of Tumor necrosis factor alpha (*Tnf-α*) using qRT-PCR in the outflow veins. The average gene expression of *Tnf-α* gene was significantly decreased in outflow veins treated with Group S compared to Group C by day 7 (Fig. 8; *P* < 0.01) demonstrating a reduction in inflammatory cytokine expression.

## Discussion

**Figure 9:**
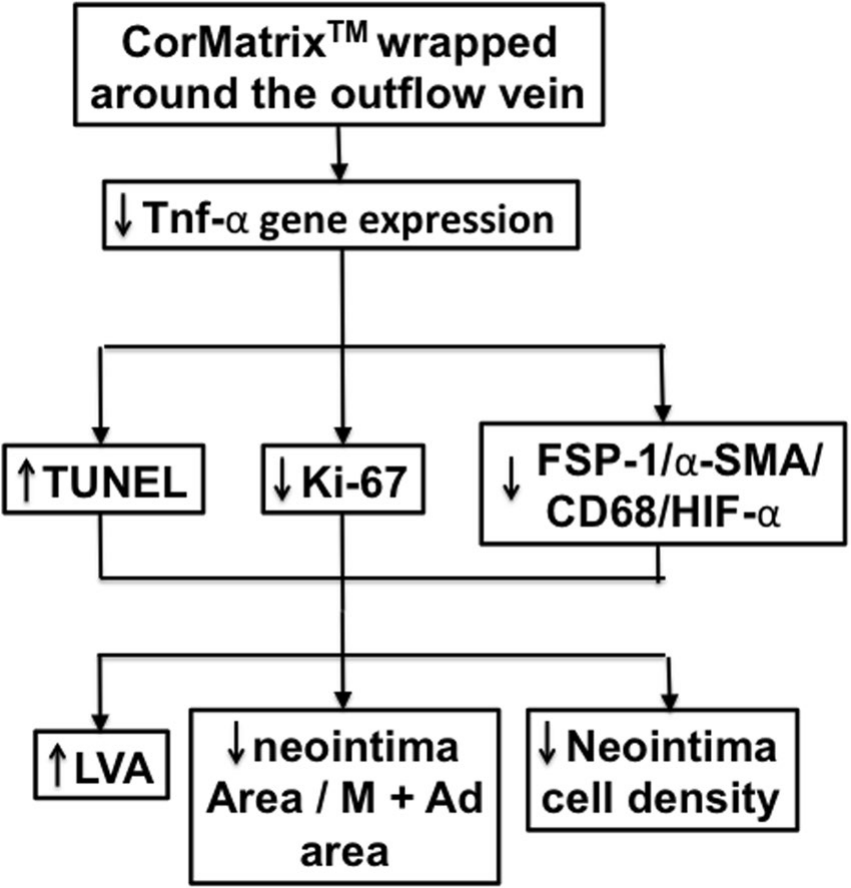
Synopsis of findings from the current study. Abbreviations: Tnf-α = tissue necrosis factor-α. TUNEL = TdT-mediated dNTP nick end labeling Ki-67 = cellular proliferation FSP-1 = fibroblast specific protein-1 α-SMA = α-smooth muscle actin CD68 = marker for macrophage HIF-1α = hypoxia inducible factor-1α. M + Ad = media + adventitia LVA = lumen vessel area.

Our results from the present study identify an important role for using CorMatrix wrap around outflow veins of AVF in reducing VNH in an experimental mouse model. The benefits of CorMatrix wrap include an increase in lumen vessel area of the outflow vein with positive vascular remodeling of the AVF with a reduction in collagen deposition. This was accompanied by an increase in apoptosis with a reduction in cellular proliferation. There is a reduction in fibroblasts, myofibroblasts, macrophages, and leukocytes accompanied with a decrease in gene expression of *Tnf-α* in outflow veins treated with CorMatrix (Fig. 9),

Recently, CorMatrix scaffold material, a decellularized extracellular matrix, has been used for cardiovascular applications^6–11^. Study performed by Kohler, *et al*. examined the effect of reducing the radius of the vessel wall acutely and thus diminishing wall stress using rigid polytetrafluoroethylene (PTFE) material and its effect on VNH. This study found that the vessel wall area and smooth muscle cell density were significantly reduced in tight-PTFE wrapped venous segments^21^. This also resulted in a decrease in the outflow vein dilation of AVF. In the present study, CorMatrix scaffold was wrapped loosely around the outflow vein of AVF reduces VNH accompanied by an increase in lumen area and results in positive outward vascular remodel.

The involvement of adventitial fibroblasts in the pathogenesis of hemodialysis vascular access dysfunction has been well described^17,18^. In the present study, a significant reduction in fibroblasts and myofibroblasts was observed in the outflow vein treated with CorMatrix compared to the control group. The results were confirmed by Western blot for α-SMA and FSP-1. We speculate that CorMatrix wrap provides a natural infrastructure for fibroblasts to migrate to and reside in, thereby reducing fibroblast migration toward the intimal region of the vessel wall.

Studies from clinical samples and pre-clinical models show that an increase in cell proliferation and a decrease in apoptosis are critical pathologic changes observed in failed hemodialysis vascular access^22–24^. This occurs when arterial blood flows into the vein, which leads to an increase in blood flow rate and interstitial pressure, which this is hypothesized to cause an increase in apoptosis^25^. Taken together, this results in an imbalance between cell apoptosis and proliferation in VNH. In the present study, we observed a significant reduction in cellular proliferation in vessels treated with CorMatrix wrap compared to controls. One of the reasons for using the scaffold wrap is that it exerts an anti-proliferative effect in the vessel wall, which may be due to a decrease in fibroblast or myofibroblast differentitation. Moreover, in addition to reduced cellular proliferation, there is a substantial increase in TUNEL staining and cleaved caspase-3 levels in scaffold-wrapped vessels indicating that CorMatrix wrap increases apoptosis in the outflow vein. Collectively, these results suggest that CorMatrix wrap modulates cell turnover in the vessel wall and thus reduces VNH.

Increased HIF-1α expression has been observed in the outflow vein of AVF in animal models and clinical specimens removed from patients of hemodialysis vascular access failure^12,19^. Hypoxic injury to the vascular wall is one of the initial factors contributing to VNH and arterial bypass graft failure^26–28^. Consistent with this, we found that HIF-1α staining was markedly decreased in the outflow veins treated with CorMatrix. This may be due to a decrease in cell density that may reduce oxygen demand in the vessel wall, resulting in decreased HIF-1α expression. Decreased HIF-1α expression may reduce the hypoxia-induced inflammatory response in the outflow veins treated with CorMatrix scaffold. One possible explanation for hypoxic injury is the disruption of vasa vasorum that supplies blood to the vessel wall during AVF creation^14^. In addition the wound healing process is a trigger for hypoxia signaling. Hypoxia signaling is known to modulate cell migration, proliferation, and differentiation resulting in VNH formation and vascular stenosis. In support of this notion, hypoxia accelerates the conversion of fibroblast to myofibroblasts (α-SMA-positive cells) and promotes proliferation and invasion of myofibroblasts^14,15^.

A decrease in inflammatory cells such as leukocytes and macrophages has been associated with improved fistula function in experimental animal models^29–31^. Anti-inflammatory molecules such as simvastatin have been shown to increase lumen vessel area in AVF outflow veins^15^. Consistent with these observations, we found a substantial decrease in macrophages and leukocytes in the vascular walls of outflow veins wrapped with CorMatrix, which was associated with reduced VNH.

To explore the molecular mechanism of CorMatrix wrap reducing VNH, gene expression of *Tnf-α* was analyzed by qRT-PCR. This demonstrated a significant decrease in the average gene expression of *Tnf-α* in outflow veins treated with CorMatrix wrap compared to control. Studies have shown that TNF-α is produced by different cell types including fibroblasts, smooth muscle cells and endothelial cells in response to hypoxia, sheer stress and inflammation^32^. Furthermore, fibroblasts tend to acquire pro-inflammatory and fibrogenic phenotype during hypoxia^33^. In the present study, vessels wrapped with CorMatrix wrap have shown a decrease in fibroblast content. The decrease in inflammatory fibroblasts may explain the low *Tnf-α* gene levels. Collectively, our results suggest the effect of CorMatrix wrap in reducing VNH may be due to the fact that the CorMatrix wrap acts as a trap for fibroblast migration and also attenuates the proliferation of fibroblast and myofibroblasts through *Tnf-α* pathway.

### Limitations

A murine model of normal kidney function was employed due to the high mortality in immunodeficient mice with chronic kidney disease, and thus the effects of chronic kidney disease could not be evaluated. Further studies should be performed to corroborate these findings using immunocompetent animals. Moreover, a larger animal model should be employed for the study.

In conclusion, this study demonstrates that utilizing CorMatrix wrap around the outflow vein in a murine model improves fistula function and reduces VNH. This is accompanied by a reduction of several cells, including fibroblasts, myofibroblasts, macrophages, and leukocytes. The present study provides a potential therapeutic approach to reducing VNH formation associated with AVF.

## Materials and Methods

### Materials

The CorMatrix scaffold material (155-µm thick with pore sizes up to 50 µm) was obtained from CorMatrix Cardiovascular, Inc. (Roswell, GA). Antibodies to FSP-1, hypoxia-inducible factor 1α (HIF-1α), and CD68 were obtained from Novus Biologicals (Littleton, CO). Ki-67 and CD45 antibodies were from Biolegend (San Diego, CA). Caspase-3 and cleaved caspase-3 antibodies were obtained from Cell Signaling Technology (Danvers, MA), and α-SMA antibody was obtained from Abcam (Cambridge, UK). Unless otherwise specified, all other reagents were obtained from Sigma-Aldrich (St. Louis, MO).

### Experimental Animals

B6.Cg-*Foxn1* ^*nu*^/J mice (20–25 g, 6–8 weeks old) were purchased from Charles River Laboratories, Inc. (Wilmington, MA). B6.Cg-*Foxn1* ^*nu*^/J mice are athymic and immunodeficient and are, therefore, an ideal strain for grafting CorMatrix. All *in vivo* procedures were conducted by following the protocols approved by the Mayo Clinic Institutional Animal Care and Use Committee. All mouse procedures were conducted according to the Public Health Service Policy on Humane Care and Use of Laboratory Animals, 2000. Mice were housed at 22 °C, 41% relative humidity, and 12-/12-hour light/dark cycles.

### Creation of the Arteriovenous Fistula with outflow veins treated with CorMatrix wrap

B6.Cg-*Foxn1* ^*nu*^/J mice (n = 46) underwent a surgical procedure for placement of an AVF as described previously^14,15,34^. The mice were anesthetized with ketamine hydrochloride (0.1–0.2 mg/g) and xylazine (0.02 mg/g) by intraperitoneal injection. The skin overlying the common carotid artery and ipsilateral external jugular vein was incised and these vessels identified and separated. All branches of the jugular vein were ligated. The common carotid artery was clamped proximally; the distal side was tied immediately below the carotid bifurcation and transected. The distal end was pulled through, inverted over, and tied to the cuff (Laboratory PolyE Polyethylene Non-Sterile Tubing, Harvard Apparatus, Holliston, MA) of the AVF. The jugular vein was ligated distally and transected. The transected end was then placed over a cuff and tied with an 8–0 Ethilon suture to form an end-to-end anastomosis between the common carotid artery and jugular vein. A piece of CorMatrix scaffold (1 × 4 mm) was wrapped around the outflow vein and sutured using 8-0 nylon to secure the scaffold to the outflow vein as shown in Fig. 1. After the surgical procedure, all animals were allowed to recover under heat lamps to maintain body temperature. Post recovery, the animals were transferred to their housing facility.

At the end of the experiment, 7 and 21 days after AVF placement, the mice were sacrificed while under anesthesia. Animals were perfused with 4% buffered formalin through the heart as described previously^15^. The outflow veins were excised under the microscope, and fixed in 4% formalin for immunohistochemistry (n = 6 per group, for both time points). Outflow veins and contralateral jugular vein were harvested and flash frozen in liquid nitrogen for Western blotting (n = 3 per group, for day 7 only). Outflow veins and contralateral jugular veins were harvested and placed in RNA later for PCR analysis (n = 6 per group, for day 7 only).

### Immunohistochemistry

Formalin-fixed blood vessels were embedded in paraffin. Commonly, 80 to 120 5-µm tissue sections are obtained from outflow vein. Histologic sectioning of tissues blocks started from the outflow vein with three tissue sections per slide. One slide from every five slides (total 5–6 slides) was used for tissue morphometric analysis. Following heat-induced antigen retrieval using antigen retrieval solution (Dako, Carpinteria, CA), tissue sections were stained with markers for cell proliferation (Ki-67), macrophage infiltration (CD68), fibroblasts (FSP-1), smooth muscle cells (α-SMA), leukocyte (CD45), and HIF-1α. Briefly, tissue sections were incubated with appropriate primary antibody in antibody diluent solution (Ki-67, 1:200; CD68, 1:200; FSP1, 1:200; α-SMA, 1:200; HIF-1α, 1:200) for one hour at room temperature. Tissue sections were then washed in wash buffer and incubated with respective HRP-conjugated secondary antibody for one hour at room temperature followed by DAB chromogen solution. For CD45 staining, tissue sections were incubated with PerCP fluorophore-conjugated CD45 antibody (1:100) overnight at 4 °C followed by DAPI counter staining. Tissue sections were also stained for respective nonspecific immunoglobulin G (IgG) antibodies to serve as negative controls.

### Verhoeff-Van Gieson Staining

To measure the formation of neointima and different layers of the vessel, VVG staining (Newcomer Supply, Middleton, WI) was performed following the manufacturer’s protocols.

### Picrosirius Red staining

To measure collagen deposition, tissue sections of AVF outflow veins at day 7 and day 21 were stained for Picrosirius red as described previously^15^.

### TUNEL Staining

The extent of apoptotic cell death in the outflow veins was measured using TUNEL staining (Trevigen, Gaithersburg, MD) following the manufacturer’s protocols. Tissue sections stained with TUNEL but without terminal deoxynucleotidyl transferase enzyme treatment served as negative controls.

### Western Blot

The outflow veins were washed in PBS and homogenized in RIPA buffer containing inhibitors for proteases and phosphatases. Each group had 3 samples that were pooled together for Western blot analysis due to low amount of protein per sample. Tissue lysates were clarified by centrifugation at 12,000 × g for 30 minutes at 4 °C. Protein content was determined using the Bio-Rad DC Protein Assay Kit (Bio-Rad Laboratories, Hercules, CA). Lysates (30-μg) were resolved on 4% to 20% gradient SDS-PAGE and then transferred to PVDF membrane following standard protocols. The membranes were probed for α-SMA, CD68, FSP-1, caspase-3, and cleaved caspase-3 (activated caspase-3).

### Real-time Polymerase Chain Reaction Analysis

The outflow vein and contralateral jugular vein were harvested and kept in RNA stabilization solution (Qiagen, Gaithersburg, MD). Samples were homogenized and the total RNA was isolated using the RNeasy Mini Kit (Qiagen). Gene expression was examined using real-time PCR analysis. Primer sequences for TNF- α included: Forward, GCTCTTCTGTCTACTGAACTTCG, Reverse, GATGAGAGGGAGGCCATTTG. PCR conditions included 95 °C for 5 min, 95 °C 15 sec, 55 °C 30 sec, 45 cycles using iTaq Universal SYBR mix in a Bio-Rad C1000 thermal cycler with CFX96 real-time system. The fold change in the gene expression was calculated following ΔΔ^CT^ method after normalizing with 18S RNA and contralateral jugular vein.

### Morphometric and Image Analysis

Image analysis was done as described previously^15^. Specifically, images of the entire cross section (5× magnification) were captured using a Carl Zeiss microscope and analyzed using Zen 2.3 digital imaging software (Zeiss, US). Immunostaining for Ki-67 (brown), α-SMA (brown), FSP-1 (brown), CD68 (brown), TUNEL positive (dark brown), and HIF-1α (brown) were selected, in turn, by selecting the appropriate red-green-blue color intensity range and then counted. The color intensity was kept the same for all sections for a particular antibody staining. Cell density as well as the neointima, media + adventitia area, and lumen vessel area were measured by manually tracing the vessel wall using the Zen 2.3 software. In the present study, the area of the neointima was identified by the internal elastic lamina, which is poorly defined in veins. In addition, the collagen deposition in the outflow vein of the AVF can be used to identify the neointima, as there is excessive collagen deposition in the neointima when compared to media and adventitia. An independent technician blinded to the experiment completed all image analysis. Slides from animals with poor tissue quality were excluded from analysis.

### Statistical Analysis

Data are expressed as mean ± SEM. The Student *t* test was conducted with post hoc Bonferroni correction. Significant differences between two groups were indicated by *P < 0.05, ^#^ *P* < 0.01, ^##^ *P* < 0.0001. JMP Pro software (SAS Institute, Inc., Cary, NC) was used for statistical analysis.

## Electronic supplementary material

Supplementary files
