# Supplementary material for: CorMatrix Wrapped Around the Adventitia of the Arteriovenous Fistula Outflow Vein Attenuates Venous Neointimal Hyperplasia
Source: Sci Rep. 2017 Oct 30;7:14298. doi: 10.1038/s41598-017-14696-z (PMC5662725; doi:10.1038/s41598-017-14696-z)
Supplement: Supplementary file 1 — Supplementary files [file 41598_2017_14696_MOESM1_ESM.pdf]

CorMatrix Wrapped Around the Adventitia of the Arteriovenous Fistula Outflow Vein Attenuates Venous Neointimal Hyperplasia

Binxia Yang, MD, PhD,[1] Sreenivasulu Kilari, PhD,[1] Akshaar Brahmbhatt, BS,[1] Deborah L. McCall, MS,[1] Evelyn Nieves Torres, PhD,[1] Edward B. Leof, PhD,[2] Debabrata Mukhopadhyay, PhD,[2] Sanjay Misra, MD[1,2]

[1] Vascular and Interventional Radiology Translational Laboratory, Department of Radiology and [2] Department of Biochemistry and Molecular Biology, Mayo Clinic, Rochester, Minnesota, USA.

Address correspondence to:

Sanjay Misra, MD, Department of Radiology, Professor of Radiology, Mayo Clinic, 200 First St SW, Rochester, MN 55905, Telephone: 507-293-3793, Fax: 507-255-7872, Email: misra.sanjay@mayo.edu.

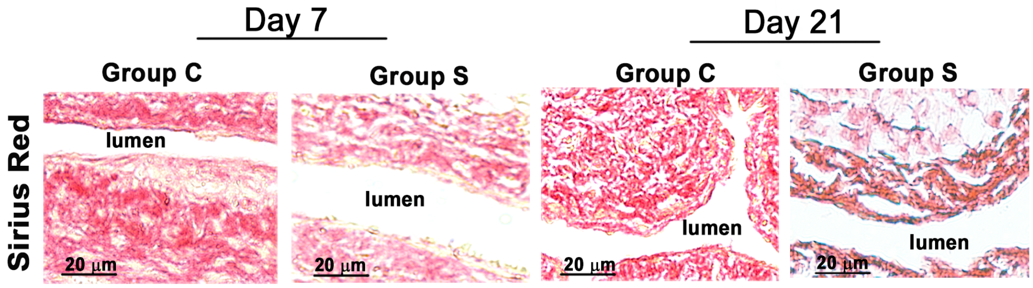

SUPPLEMENTARY Figure S1: Picrosirius red staining of AVF outflow veins wrapped with CorMatrix scaffold. Picrosirius red Staining of outflow vein (Group C) and CorMatrix outflow vein (Group S) sections at day 7 and day 21 post fistula creation. The more intense red staining is representative of collagen 1 and 3 staining. Qualitatively, there is decreased Sirius red staining in Group S compared to Group C. All images were taken at 100× magnification and the scale bar indicates 20μm.

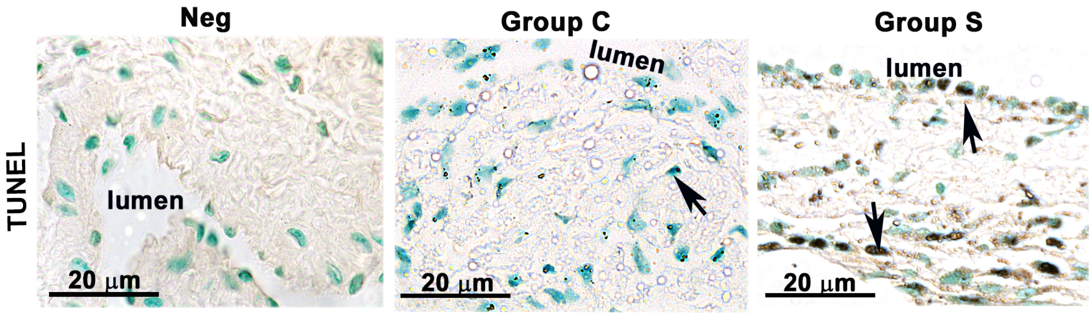

SUPPLEMENTARY Figure S2: Assessment of cell apoptosis in the AVF outflow veins wrapped with CorMatrix scaffold. Cell death was assessed using TUNEL on AVF tissue sections at days 21 after fistula placement. TUNEL staining at day 21 outflow veins without (Group C) or with (Group S) CorMatrix wrap. The first column is the negative control .Dark brown nuclei positive for TUNEL are indicated by arrows. All images were taken at 100× magnification and the scale bar indicates 20 μm.
